# A Genus Comparison in the Topological Analysis of RNA Structures

**DOI:** 10.1007/s10441-025-09500-9

**Published:** 2025-08-01

**Authors:** Nicolò Cangiotti, Stefano Grasso

**Affiliations:** 1 Department of Mathematics, Politecnico di Milano, via Bonardi 9, Campus Leonardo, Milan, 20133 Italy; 2 Lesaffre International, 101 Rue de Menin, Marcq-en-Barœul, 59700 France; 3 Department of Medical Microbiology, University Medical Center Groningen, University of Groningen, Hanzeplein 1, PO Box 30001, 9700 RB Groningen, The Netherlands

**Keywords:** RNA folding, Genus, Pseudoknots, RNA tertiary structure, Feynman diagrams, 82D60, 81T18

## Abstract

While RNA folding prediction remains challenging, even with machine and deep learning methods, it can also be approached from a topological mathematics perspective. The purpose of the present paper is to elucidate this problem for students and researchers in both the mathematical physics and biology fields, fostering interest in developing novel theoretical and applied solutions that could propel RNA research forward. With this intention, the mathematical method, based on matrix field theory, to compute the topological classification of RNA structures is reviewed. Similarly, McGenus, a computational software that exploits matrix field theory for topological and folding predictions, is examined. To further illustrate the outcomes of this mathematical approach, two types of analyses are performed: the prediction results from McGenus are compared with topological information extracted from experimentally-determined RNA structures, and the topology of RNA structures is investigated for biological significance, both in evolutionary and functional terms. Lastly, we advocate for more research efforts to be conducted at the intersection between physics, mathematics and biology, with a particular focus on the potential contributions that topology can make to the study of RNA folding and structure.

## Introduction

In the last decade RNA science boomed, consequently, the range of its applications drastically increased, even more with the recent advent of synthetic biology (Dykstra et al. 2022). RNA can perform a diverse set of functions, which are already exploited for commercial applications, such as innate immunity modulation, chemical reactions catalysis, small molecules sensing, and gene expression regulation (Dykstra et al. 2022; Townshend et al. 2021).

RNA is a single-filament polymer, whose monomers are ribonucleotides. In turns, ribonucleotides are made of a ribonucleoside and a phosphate group, which bind each monomer to the adjacent one. Ribonucleosides are formed by a sugar (*i.e.* the ribose) and nucleobase (also called nucleotide base or nitrogenous base). Four main nucleobases are present within the RNA molecule, forming the four main ribonucleosides: adenosine (A), cytidine (C), guanosine (G) and uridine (U). Across the different domains of life, nucleobases can be post-transcriptionally modified resulting in more than 100 different ribonucleosides, the most known being inosine (I), generated from A, and pseudouridine ($\Psi$) and dihydrouridine (D), both modified from U (McCown et al. 2020). Despite being single-filament, and similarly to DNA, RNA bases can form bonds with bases from other molecules, or more interestingly in this context, also within the same molecule, thus creating complex 2D and 3D structures. In the canonical Watson-Crick base-pairings (cWW), A binds U with two hydrogen bonds, and G binds C with three hydrogen bonds. RNA molecules also exhibit non-canonical base pairings, with the G:U bond being the most notorious (Das et al. 2006). Due to the didactic approach of this paper, the consideration of all possible modified bases and non-canonical pairings is intentionally omitted for simplicity, yet they have a significant impact on RNA structure. This is particularly relevant, since what determines a RNA molecule’s behavior and function is, in large proportion, its complex structure.

**Fig. 1:**
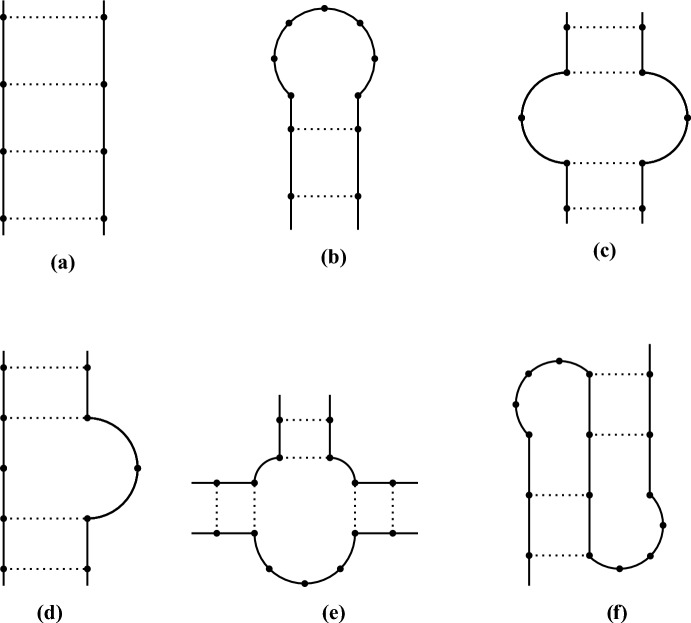
Classical *ladders and loops* representations of various RNA secondary structures mentioned in the text, and the *pseduoknot*. In this type of representation, the solid line represents an RNA molecule, with each dot along it indicating a ribonucleoside, and dotted lines depicting hydrogen bonds between the canonical Watson-Crick base-pairings (cWW). In (**a**) a stem is shown, the only structure having all bases engaged in a bond. The other figures represent structures consisting of free bases only, but some stems have been depicted as well for clarity: (**b**) hairpin-loop and (**c**) internal-loop are both examples of loops; (**d**) bulge; (**e**) junction; (**f**) pseudoknot. While a planar representation of the pseudoknot is possible, this does not consider the physical constraints of the RNA molecule, which, in reality, occupies a 3D space

RNA structural motifs determine and stabilize RNA secondary structure, and are made up of two components: the first one consisting of stems (or helices), *i.e.* stretches of an RNA molecule with all the bases engaged in forming a hydrogen bond (Fig. 1a); and the second one made up of free bases like in loops (Fig. 1b and 1c), bulges (Fig. 1d), and junctions (Fig. 1e). Elements of each component are thus alternated, *i.e.* motifs composed of paired bases are alternated to motifs with free bases, giving rise to complex RNA secondary structures (Antczak et al. 2017). Secondary structures from different parts of the molecule tend to interact with each other, folding hierarchically to form the tertiary structure, composed of more complex elements. The most interesting and important of them, especially within the context of the present paper, is the *pseudoknot* (Fig. 1f). A *pseudoknot* is composed of at least two helices with both internal bonds and free bases interacting across the two motifs, and being separated by a additional stretch of free bases (Dam et al. 1992). Because of this, *pseudoknots* are made of simpler interacting motifs that raise RNA planar structure to a 3D one, making RNA molecules more compact, yet more complex. Basically, RNA secondary structure represents its planar conformation, while the tertiary structure is its 3D conformation, and ultimately determines its specific function (Antczak et al. 2017).

While RNA secondary structure is generally understood and can be predicted with a sufficient degree of accuracy (Fallmann et al. 2017), the same cannot be said, despite the efforts, for RNA tertiary structure (Townshend et al. 2021; Weeks 2021). However, to fully harness the potential of RNA molecules as biotechnological tools by engineering them, and because there is an intimate link between its structure and function, there is an increasing need to predict its folding. In this task, assistance can be provided by experimental data, which is still lacking at the moment and, unfortunately, remains hard to collect (Dykstra et al. 2022; Townshend et al. 2021; Zhang et al. 2022). This advocates for more experimental efforts (Dykstra et al. 2022; Townshend et al. 2021) and more understanding of the underlying physics of RNA folding. In fact, such data could be used to train novel machine learning (ML) models, which are now able to combine large (experimental) data sets with physical constrains, in order to achieve better predictions (Karniadakis et al. 2021).

Interestingly, RNA folding can be addressed using matrix field techniques originated from quantum field theory^1^. To this aim, we shall review how the interactions between ribonucleotides can be approached combining the expansion of the partition function of RNA folding with the combinatorial terms of *Feynman diagrams*, and then translate it in terms of matrix field theory (Orland and Zee 2002). It turns out that *pseudoknots*, *i.e.* one of the motifs composing the RNA tertiary structure, play a key role in RNA topological behaviour. Within this context, the concept of *RNA genus* was introduced exploiting the parallel with mathematical topology and combinatorial maps^2^.

The aim of the present paper is twofold. Firstly, to introduce the diagrammatic approach based on QFT to students and researchers from different fields approaching for the first time the not yet fully elucidated problem of RNA structure and its prediction. Secondly, the paper aims at fostering research across the mathematical physics and biology fields, boosting the development of this fascinating theory. With this trajectory in mind, starting from Orland and Zee (2002), the key concepts of the topological theory are summarized by surveying its theoretical and practical applications towards the RNA molecule. Subsequently, the biological meaning of the genus is investigated by examining a dataset of RNA sequences from both a taxonomical and functional point of view.

## Physical Background: A Review

In 2002, Orland and Zee (2002) introduced a very intriguing method for predicting the tertiary structure of RNA sequences based on the *matrix field theory*. In particular, they noticed a profound analogy between the model obtained by pulling, from its extremities, the classical *ladders and loops* visualization diagram of RNA secondary structure (*e.g.* those depicted in Fig. 1) and the famous Feynman diagrams, defined in quantum field theory and then also applied successfully in quantum chromodynamics (QCD). This approach is intimately connected with the topology of such diagrams, as proved by ’t Hooft (1974). This context allows for the introduction of the notion of *genus*, which can be used to topologically classify RNA structures (Vernizzi et al. 2005, 2016, 2006). In the present section, some of the most significant mathematical and physical concepts of this method are retraced, stressing the transfer from the domain of physics to the domain of biology. In a more abstract framework, the construction explained below can be seen as a particular case of the so-called *map enumeration problem*, belonging to the theory of *dessin d’enfant*^3^. This kind of problem has been extensively studied, resulting in a rigorous mathematical formulation of the topic. One of the most useful *hitchhiker’s guides* in this regard is the work of Zvonkin (1997), which clearly reviews the basics of the relation between matrix integrals and the map enumeration problem. In the next paragraphs, we shall use the formalism originally used by Orland and Zee (2002) with the aim of preserving the connection with the analyses performed in Sect. 3 with McGenus software.

Referring back to the biological context, the RNA energy models studied in recent years are represented through a so-called partition function $\mathcal {Z}$. From a biophysical perspective, $\mathcal {Z}$ represents the statistical sum over all possible conformational states of the RNA molecule, and encodes the complete thermodynamic information needed to predict its folding probabilities and structural properties at thermal equilibrium. In the three-dimensional case, for a sequence of length *L*, $\mathcal {Z}$ is given by^4^

1 $$\begin{aligned} \mathcal {Z}=\int \prod _{k=1}^L \text {d}^3\textbf{r}_k f(\textbf{r})Z_L(\textbf{r})\,, \end{aligned}$$

where $\textbf{r}_k$ is the 3D position vector of the *k*-th nucleobase, and $f(\textbf{r})$ is a model-dependent function that accounts for various physical properties of the RNA chain, such as its geometry, stiffness, and steric constraints^5^.

In particular, the function $Z_L(\textbf{r})$ provides the description of interacting base pairs and it is constructed as follows:

2 $$\begin{aligned} \displaystyle Z_L=1+\sum _{\langle i,j\rangle }V_{ij}(\textbf{r}_{ij})+\sum _{\langle i,j,k,l\rangle }V_{ij}(\textbf{r}_{ij})V_{kl}(\textbf{r}_{kl})+\cdots \,, \end{aligned}$$

where $\langle i,j\rangle$ denotes the pair relation $j>i$, $\langle i,j,k,l\rangle$ the quadruplets relation $l>k>j>i$, and so on. The function $V_{ij}(\textbf{r})$ is the Boltzmann factor with energy $\epsilon _{ij}$ that relates the *i*-th and the *j*-th base at the distance $\textbf{r}_{ij}=\vert \textbf{r}_i-\textbf{r}_j\vert$:

3 $$\begin{aligned} V_{ij}(\textbf{r}_{ij})=\exp \left( -\beta \epsilon _{ij}s_{ij}(\textbf{r}_{ij})\right) \,, \end{aligned}$$

where $\beta = 1/(k_BT)$ is the usual symbol for the inverse temperature multiplied to the Boltzmann constant, $\epsilon _{ij}$ is the interaction energy between the *i*-th ribonucleotide and *j*-th ribonucleotide, and $s_{ij}(\textbf{r}_{ij})$ represents the space-dependent part of the interaction. Assuming infinite flexibility for the chain, sterical constraints can be neglected. One can drop all spatial degrees of freedom and obtains the simplified expression (Orland and Zee 2002; Vernizzi et al. 2005):

4 $$\begin{aligned} Z_L=1+\sum _{\langle i,j\rangle }V_{ij}+\sum _{\langle i,j,k,l\rangle }V_{ij}V_{kl}+\cdots \,, \end{aligned}$$

where now $$V_{ij}=\exp \left( -\beta \epsilon _{ij}\right)$$.

In the following sections, the focus is on the approach developed by Orland and Zee and then expanded in the next years by their collaborators (Bon et al. 2008; Orland and Zee 2002; Vernizzi et al. 2016, 2006). This choice, as aforementioned, is determined by the usage of software (McGenus) for the analysis of Sect. 4. However, for the sake of completeness, other works in similar directions should be mentioned. Strictly related to the theory introduced by Orland and Zee, we mention the work of dell’Erba and Zemba (2009), where an exact expression for the topological expansion of the partition function of the system in Orland and Zee (2002) is derived, and the work of Garg and Deo (2009), where the authors compute the exact expression of the partition function for an analogous system obtained from Vernizzi et al. (2005) with the addition of an external interaction term. Furthermore, another of the first approaches in such a direction is due to Penner and Waterman (1993). They connected RNA structures with topology by studying the space of RNA secondary structures, and proving that the geometrical realizations of the associated complex of secondary structures is a sphere. Recently, Penner has applied some mathematical objects coming from algebraic geometry, such as moduli spaces, in the study of biological macromolecules (Penner 2016). In the last few years, Andersen et al. (2013a, 2013b) proposed an analogous investigation on RNA pseudoknots based on their topology and the concept of *genus* naturally associated to the biological structures. The concept of genus was recently generalized and applied to provide a more in-depth characterization of biomolecules and their 3D structural complexity (Rubach et al. 2019; Zając et al. 2018), and further discussed in the next sections. Lastly, in Xu and Chen (2020), the authors suggested a quantitative analysis of the topological constraints on RNA three-dimensional conformational space for the distribution of helix orientations, for both loop-loop kissing structures and pseudoknots.

A similar framework in the topological analysis of RNA involving genus and pseudoknots is provided by the study of generating functions for the so-called $\gamma$-structures. Succinctly said, a $\gamma$-structure is a cross-free, arc-labeled structure consisting in a specific building of blocks, that all have topological genus less than or equal to a given value, namely $\gamma$. In particular, we remark that these structures are actually pretty different from the classical pseudoknotted RNA structures of fixed topological genus (associated, for instance, to a fatgraph or double line graph). Indeed they don’t have a fixed genus, but they are composed by irreducible subdiagrams whose individual genus is bounded by $\gamma$, and contain no bond of length one. In Li and Reidys (2013), Li and Reidys derive a new bivariate generating function for $\gamma$-structures by using different mathematical techniques such as symbolic methods, the singularity analysis of the solutions, bivariate polynomials, and a central limit theorem. In particular, they showed that the average genus for random 1-structures (obtained via nesting and concatenating structures of genus at most 1) satisfies a central limit theorem. Surprisingly, as reported in Sect. 4, our analyses find values similar to the theoretical ones given in Li and Reidys (2013). The same authors (with Han) also proposed a more in-depth study about the combinatorics of $\gamma$-structures using irreducible shadows (Han et al. 2014). Additionally, they extended the analysis by deriving a new bivariate generating function to investigate the distributions of arcs, stacks, hairpin-, internal- and multi-loops, and H-type pseudoknots (Li and Reidys 2017).

Finally, the existence of another interesting method called trisection, based on a combinatorical and topological manipulation, should be highlighted. It was introduced by Chapuy in (2011) while exploring the so-called unicellular maps: graphs embedded in an orientable surface, such that their complements are topological disks. In his analysis, he offered a new point of view to the structure of these particular objects, decomposing any unicellular map into a unicellular map of smaller genus. An interesting development in the framework of bijections based on topological trisections was proposed by Huang and Reidys in (2016). The authors presented a first context-free grammar for RNA pseudoknot structures with the feature of generating any RNA structure (including pseudoknot structures).

### Behind the Origin of the Diagrams

In the spirit of this survey section, it is important to notice that the fundamental role in the work of Orland and Zee (2002) is played by the following kind of integrals over the space of $N \times N$ Hermitian matrices^6^:

5 $$\begin{aligned} \begin{aligned} Z_n(a,N)=&\frac{1}{A(N)}\int \textrm{d}^{N\times N}\phi \exp \left( -\frac{N}{2a}{{\,\textrm{Tr}\,}}{\phi ^2}\right) \\&\times \frac{1}{N}{{\,\textrm{Tr}\,}}\left( {1}+\phi \right) ^n, \end{aligned} \end{aligned}$$

where the normalization factor *A*(*N*) is a function depending on *N*. Here $\phi$ is an Hermitian matrix, namely it is equal to its transpose conjugate, and ${{\,\textrm{Tr}\,}}(\cdot )$ represents the trace operator. To better understand the significance of such integrals and the link with the diagrams picture (Bouttier 2011), it could be helpful to consider the following one-dimensional Gaussian integral, which actually represents the evaluation of average values of the function $(1+x)^n$ with respect to the Gaussian weight (Vernizzi et al. 2005):

6 $$\begin{aligned} Z_n(a)=\frac{1}{A}\int _{\mathbb R} \textrm{d}x \exp \left( -\frac{x^2}{2a}\right) (1+x)^n\,, \end{aligned}$$

where *A* is the following normalization constant

7 $$\begin{aligned} A=\int _{\mathbb R} \textrm{d}x \exp \left( -\frac{x^2}{2a}\right) =\sqrt{2\pi a}\,. \end{aligned}$$

**Fig. 2:**
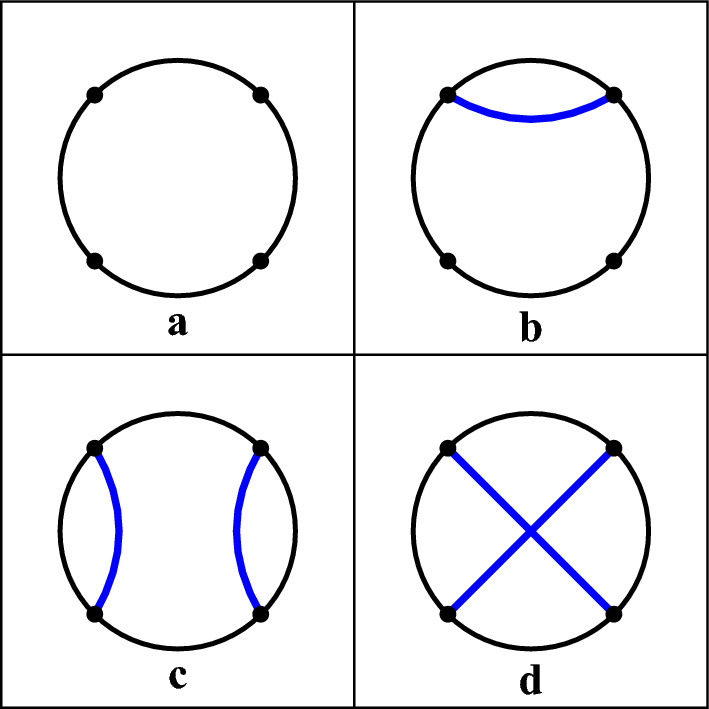
Four examples of diagrams representing terms of the integral $Z_4(a)=1+6a+3a^2$. In the Figure are shown: (**a**) the no-chords diagram, *i.e.* the addendum 1; (**b**) a one-chord diagram contributing to the first order term 6*a*; two of the three second order two-diagrams in $3a^2$: one planar (**c**) and one non-planar (**d**). In the RNA context, each point on the circle represents a nucleotide base in the sequence, and crossing chords correspond to a pseudoknot, since they indicate base pairings that cannot be drawn without intersection in the planar representation. This graphical criterion reflects the standard operational definition of pseudoknots within the context of RNA secondary structures: the presence of at least two helices whose base pairings are topologically entangled. Although a crossing does not fully capture the three-dimensional details of the molecule, it reliably signals the minimal topological complexity required to go beyond planar configurations, justifying its use as a proxy for tertiary interactions such as pseudoknots

Here, the number $a\in \mathbb {R}_{>0}$ is called, by analogy with quantum theory, *propagator*. It is now possible to provide a useful interpretation of these integrals in terms of diagrams, which, for historical reasons, are called *Feynman diagrams*. Taking, for instance, $n=2$ in (6), one immediately gets (after a few computations) $Z_2(a)=1+a$, where, considering a circle and two points on it, the addendum 1 stands for the so-called no-chords diagram, and the term *a* stands for the diagram with chords joining the two points. In the same way, it is possible to compute also $Z_4(a)=1+6a+3a^2$, where, in addition to the no-chords diagram, there are also 6 one-chord diagrams represented by the term 6*a*. Finally, $3a^2$ are the 3 possible combinations of two-chords diagrams. Fig. 2 shows some standard examples of the operation of these diagrams in one-dimension. However, it is important to underline that these one-dimensional integrals provide only combinatorial informations about the number of possible chord diagrams, without distinguishing between planar and non-planar configurations. In the following lines we shall discuss how the $N\times N$ matrix theory is essential to obtain refined topological informations.

The power of this approach is clearly visible when one considers Eq. (5) for large value of *N*, namely the large-*N* expansion. Indeed, the computation of $Z_2(a,N)=1+a$ does not depend on *N*, but computing $Z_4(a,N)=1+6a+2a^2+a^2/N^2$ one explicitly obtains the dependence on *N*. Overall, Eq. (5) can be computed by using the renowned *Wick theorem*^7^. The application of the latter theorem produces a function of *N*, which can be represented as an asymptotic series:

8 $$\begin{aligned} Z_L=1+\sum _{\langle i,j\rangle }V_{ij}+\sum _{\langle i,j,k,l\rangle }V_{ij}V_{kl}+\frac{1}{N^2}\sum _{\langle i,j,k,l\rangle }V_{ik}V_{jl}+\cdots . \end{aligned}$$

This link between Eq. (4) and (8) is clear: the two series are exactly the same for $N=1$, but for $N>1$ Eq. (8) also carries some topological information. In particular, it should be remarked that the first order gives all the planar structures, while second-order terms actually represent the RNA secondary structures and pseudoknots. In other words, the term involving $1/N^2$ represents, in diagrammatic terms, the non-planar diagram: the one with the intersecting chords, *i.e.* the diagram (*d*) in Fig. 2. In Orland and Zee (2002), the authors exploit the analogy between Feynman diagrams and disk diagrams (see also Fig. 4b), used as representation of RNA secondary structure, to study the possible pairings within RNA molecules. Indeed, the higher-order terms in the expansions correspond to RNA secondary structures with pseudoknots. Thus, an in-depth analysis can help to tackle the problem of RNA-folding prediction.

The analogy with Feynman diagrams provides more than a visual intuition: it offers a powerful combinatorial framework to represent and analyze RNA folding configurations. As we have explained, by mapping base pairings to chord connections and interpreting crossing patterns as structural entanglements, one can translate the problem of RNA structure prediction into the language of perturbative expansions. In practice, each term in the series corresponds to a class of pairing configurations, and the graphical representation makes it possible to enumerate and classify them systematically. This approach, inherited from matrix models in quantum field theory, enables tractable computations over vast configuration spaces, which are otherwise intractable in direct molecular simulations.

However, it must be underlined that this analogy also has limitations. While chord diagrams capture the connectivity of base pairings, they do not encode geometric constraints, chemical environments, or kinetic effects that play essential roles in the actual folding process. A crossing in the diagram indicates topological complexity, but it does not uniquely determine the three-dimensional conformation or functional viability of the structure. As such, the analogy is best understood as a structural skeleton: a global, topology-driven outline of folding possibilities, which must eventually be complemented by more detailed energetic and spatial modeling to achieve predictive accuracy.

In the next Section, the topological characterization of these graphs is explored. In particular, the *genus* (Orland and Zee 2002), a fundamental topological concept strictly correlated to the notion of pseudoknot, is revised. A more recent research about genus ranges of chord diagrams can be found in Burns et al. (2015).

### The Link Between Genus and Pseudoknots

The usage of diagrams to describe RNA secondary and tertiary structures, thus also pseudoknots, is nowadays a practice, as testified by a large number of representations found in the literature as one can see, for instance, in Andersen et al. (2013a, 2013b); Orland and Zee (2002); Pillsbury et al. (2005); Rubach et al. (2019); Vernizzi et al. (2005, 2006); Xu and Chen (2020); Zając et al. (2018).

**Fig. 3:**
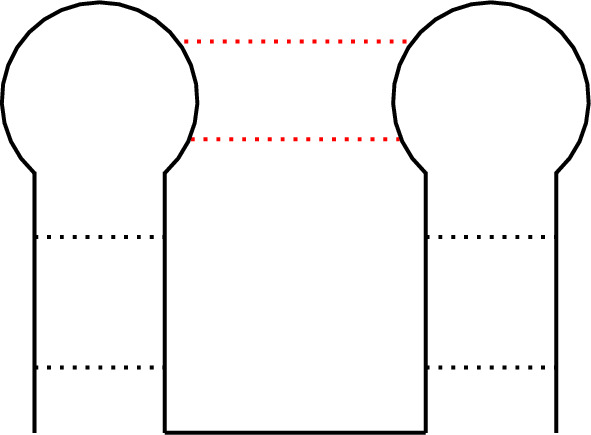
Classical diagram of a *kissing hairpin* pseudoknot

**Fig. 4:**
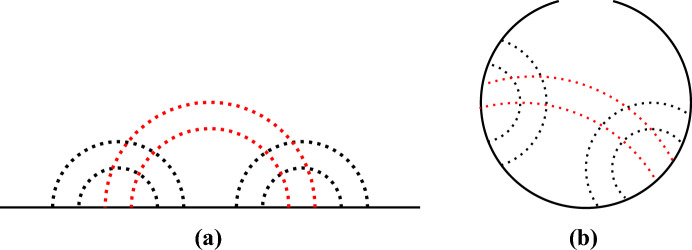
Stretching (or arc) diagram of a *kissing hairpin* pseudoknot (a). Disk diagram of *kissing hairpin* pseudoknot (b)

To exemplify this, a standard type of pseudoknot called the *kissing hairpin* (Bon et al. 2008) will be used. Its classical *ladders and loops* representation (Fig. 3), which includes the base pairs interactions, can be used as a starting point to derive, by stretching the backbone pulling from its extremities, two other useful (and topologically equivalent) depictions, namely the *stretching* (or *arc*) *diagram* (Fig. 4a) and the *disk* (or *circle*) *diagram* (Fig. 4b).

It is important to underline that the lines associated to the base pairings have to belong to the *same side* of the diagram (the upper or lower side for the stretching diagram and inside or outside of the *circle* for the disk diagram). The aim of this section is to highlight the topological connection between crossing diagrams and pseudoknots. A common way to face such a problem is to consider the diagram drawn by using the *double line notation* as illustrated in Orland and Zee (2002). However, for the purpose of this work, it is sufficient to grasp the intuitive underlying idea.

**Fig. 5:**
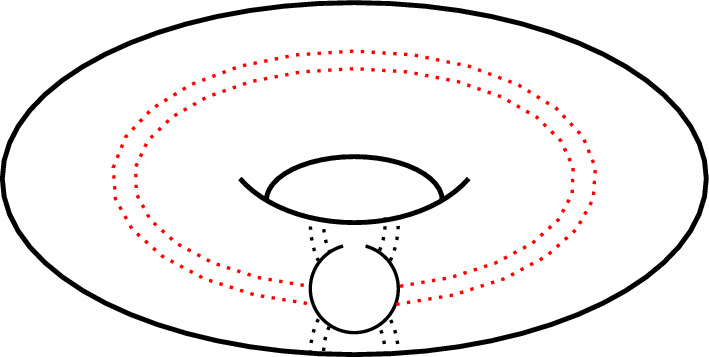
*Kissing hairpin* pseudoknot embedding on a torus. The disk diagram can be actually drawn without any crossings. This corresponds to the topological genus of the torus, namely $g = 1$ (adapted from Vernizzi et al. (2004))

The pseudoknots characterization in terms of crossing diagrams is well expressed by the *genus* of a surface. Topologically speaking, the genus of a surface is the number of *holes* and *handles* of a (orientable) surface. In this framework, the genus of a diagram can be defined as the genus of the surface with the lowest genus (*i.e.* number of *holes*) in which our diagram can be drawn without intersections. In the case of the kissing hairpin, as one can see in Fig. 5 (adapted from Vernizzi et al. (2004)), this is possible in a surface with genus $g=1$, namely a torus.

Thanks to this topological perspective it is possible to classify pseudoknots by rewriting the series expansion defined in the previous section. Indeed, in Vernizzi et al. (2004, 2006) the genus *g* is included explicitly from the original model (Orland and Zee 2002), by considering power series with respect to terms of the form $N^{-2g}$ (here *N* denotes, as above, the dimension of the matrix^8^). A practical way to compute the genus of a diagram comes from the celebrated Euler characteristic $\chi$ that in the case of diagrams is defined as $\chi =V-E+F$, where *V*, *E*, and *F* are the numbers of vertices, edges, and faces respectively. In this description, a vertex is a ribonucleotide, an edge is any line connecting two ribonucleotides, and a face is a part of the surface within a closed loop of edges. Let us suppose that the RNA secondary structure admits pseudoknots, as in the case of a kissing hairpin, the computation of the Euler characteristic leads^9^ to the value $\chi =-1$. The geometrical significance of such a value is strictly related to the number of *holes* (or *handles*) of a surface. In particular, for an orientable surface one obtains $\chi =2-2g-p$, where *p* is the number of punctures. Thus, the kissing hairpin pseudoknot induces a genus $g=1$, and can be drawn without crossing on a surface with one *hole*, that is, as said before, a *torus* (see Fig. 5). The interested reader can find a more detailed investigations on genus and pseudoknot in this context in *e.g.* Bon and Orland (2010); Bon et al. (2008); Vernizzi et al. (2005, 2016, 2006). Thanks to these topological interpretations and some considerations around statistical mechanics models of RNA, a computational implementation of a powerful algorithm has been proposed in Bon et al. (2012); Bon and Orland (2011), and will be described in more details in the next part of the paper.

In recent years, two interesting generalization of the concept of genus were introduced (Rubach et al. 2019; Zając et al. 2018), which we summarize for the sake of completeness. The first one is the *genus trace*, a function $g(i):\mathbb {N}\rightarrow \mathbb {N}$ providing the genus of a segment of the chain between the first and the *i*-th residue. The second one is instead the *fingerprint matrix*, which gives a useful mathematical visualization of all the genuses computed between two elements of a chain, namely if one uses the notation $\textbf{G}=(g_{ij})$ for the matrix, the generic element $g_{ij}$ represent the genus of the sub-chain between the *i*-th and the *j*-th residue.

## Methods: New Frontiers for McGenus

In the previous section, an in-depth review of the topological description strictly related to the large-*N* matrix field theory was provided. In Bon et al. (2012); Bon and Orland (2011); Vernizzi et al. (2004), such a framework has been adapted to develop a software for modelling RNA structure. For the purpose of this work, it seems appropriate to briefly recall the main mathematical properties of the software, which is based on the theory explained in the previous sections. Indeed, it is possible to rewrite the partition function from Eq. (1) with a more comfortable notation, highlighting some fundamental terms involved in the free energy contribution. In particular, following (Vernizzi et al. 2004), one considers the following partition function

9 $$\begin{aligned} \mathcal {Z}=\sum _{C_{ij}}\exp \left[ -\frac{1}{k_BT}\left( E(C)+T\cdot S(T,C)\right) \right] , \end{aligned}$$

where *E*(*C*) is the energetic contribution, *S*(*T*, *C*) is the entropic contribution, *T* is the temperature, and *C* is the $L\times L$ *contact matrix*, whose elements are

$$\begin{aligned} C_{ij}={\left\{ \begin{array}{ll} 1 & \text { if } i \text { and } j \text { are paired }\,;\\ 0 & \text { if } i \text { and } j \text { are not paired }\, , \end{array}\right. } \end{aligned}$$

so that the sum over $C_{ij}$ is the sum over all possible contact matrices for a given primary structure. It is important to notice that the terms involved in the Eq. (9) have been determined empirically and they are called *Turner energy rules* (Serra and Turner 1995; Zuker et al. 1999). The contribution of the topological fluctuations due to the pseudoknots, as the kissing hairpin mentioned in Sect. 2.2, can be involved into the equation as follows

$$\begin{aligned} \mathcal {Z}=\sum _{C_{ij}}\exp \left[ -\frac{1}{k_BT}\left( E(C)+T\cdot S(T,C)+\mu \cdot g(C)\right) \right] , \end{aligned}$$

where $\mu$ stands for the *topological chemical potential* and *g*(*C*) is the genus of the configuration associated to the contact matrix *C*. The relation between $\mu$ and the order *N* of the matrix formulation provided in Sect. 2 is given by the following equation (Vernizzi et al. 2004)

$$\begin{aligned} \mu =-2k_BT\log (N). \end{aligned}$$

In the next paragraphs, the data and methodologies employed in our analyses are described. The focus is on the software McGenus (Bon et al. 2012), which is based on the topological characterization described above, and on the ‘Genus for biomolecules’ database (Rubach et al. 2019), which contains genus values based on experimental RNA structural data.

### McGenus

The present comparative analysis is based on McGenus software (v7.0, August 2011, an online version available also at: https://tt2ne.ipht.fr/mcgenus.php), which is in turns rooted on algorithms exploiting the theoretical framework presented in the previous sections. McGenus is extensively described in Bon et al. (2012) and it represents an important development from its precursor TT2NE (Bon and Orland 2011). For the sake of completeness, the main features of the software are shortly summarized in the next lines. Both McGenus and TT2NE start from the same energy function, which is defined in terms of stem-like structures (helices possibly comprising bulges of size 1 or internal loops of size $1\times 1$) called *helipoints*, which minimizes the free energy. In both cases it is added the genus penalty defined above, so that the free energy $F_S$ of a given RNA structure *S* can be written as

$$\begin{aligned} F_S=\sum _{i=1}^N\sigma _i^S\cdot \Delta F(h_i)+\mu \cdot g(S), \end{aligned}$$

where $\sigma _i^S$ is an indicator function that takes on the value 0 or 1 whether helipoint $h_i$ does not or does belong to *S* respectively, $\Delta F$ is a local term indicating the free energy of individual helipoint and $\mu \cdot g(S)$ is the genus penalty term introduced before. However, while TT2NE uses a deterministic order for adding or removing helipoints in the construction of the secondary structure, McGenus adds or removes them one at a time, based on a stochastic Monte Carlo (MC) Metropolis scheme. It is important to note that there is no restriction on the pseudoknot topology that McGenus can generate.

The main limitations of these 2 tools are the maximum length of admitted sequences, up to 1000 bases for McGenus and up to 200 bases for TT2NE. And the fact that both tools can only account for the canonical cWW, thus underestimating the number of possible bonds. Further information on the algorithms can be found in Bon et al. (2012); Bon and Orland (2011). For such reasons the dataset had been generated and curated as described in Sect. 3.2.

### Dataset

*Genus* data (trace and total genus, the latter used in our comparisons), calculated on experimental structural information, were downloaded from the ‘Genus for biomolecules’ database (http://genus.fuw.edu.pl, accessed on 22nd March 2022) (Rubach et al. 2019). In the ‘Genus for biomolecules’ database 1575 unique RNA structures, used in Zając et al. (2018) to compute the genus and its derivatives, were present. Of those 1575 unique RNA structures, we discarded those whose sequence was not unique (an RNA sequence can be associated with multiple structures, *e.g.*, different conditions). Additionally, due to the limitations explained in Sect. 3.1, sequences with length higher than 1000 ribonucleotides were discarded, remaining with 739 unique sequences. Due to the same limitations, these were reported in their canonical form (*i.e.* only A, C, G, U), thus ignoring the many non-canonical bases incorporated in such molecules.

We further expanded this dataset with an additional 408 RNA sequences defined as those deposited in RNAcentral release 22 (Consortium 2020), and associated with a PDB identifier (i.e., an identifier for the Protein Data Bank). Sequences were thus downloaded from PDB (Berman et al. 2000). Again, because of the technical limitations of McGenus, outlined in Sect. 3.1, we excluded sequences longer than 1000 ribonucleotides. For the same reason, throughout this part of the dataset composed of 408 sequences, we performed a ribonucleotide replacement, substituting N (*i.e.* unspecified or unknown nucleoside), X (*i.e.* xanthosine), F (*i.e.* fluorouridine), and M (*i.e.* adenosine or cytidine) with A, and substituting V (*i.e.* either adenosine, cytidine, or guanosine) with G.

For the whole dataset, thus consisting of 1147 unique sequences, taxonomical and RNA type metadata were retrieved using the EBI search API (Madeira et al. 2022); taxid numbers were then converted into respective names using the Python package TaxidTools v. 2.2.3 (Denay 2021). When TaxidTools was not able to retreive the taxonomic information associated with a sequence, in most cases due to synthetic constructs, the label “unknown” has been assigned to all taxonomical information. The whole dataset (together with the results presented in Section 4) is available at: https://github.com/grassoste/genus-comparisons.

### Code and Analyses

Scripts to perform this study were developed in-house and written in Python.

Genus predictions were performed with McGenus option-nsuboptimal set to 10, and-maxgenus set to a value equal to the total genus trace as calculated by Rubach et al. (2019) $+5$ for the initial 739 sequences. Because for the remaining 408 sequences no reference value was available,-maxgenus was set to 5. Out of this latter set, 30 sequences showed an average genus (*i.e.* the average genus of the 10 suboptimal structures) of 4 or higher and were thus re-run with a-maxgenus of 15. Because McGenus treats RNA structures as statistical ensembles, and 10 suboptimal structures are returned, the mean of the 10 structures was used. Obviously non-integer numbers of genus do not have a counterpart in the mathematical realm, yet we believe that in this way we better capture the molecular dynamics of RNA sequences.

Calculations of the parameters of the regression equations were performed with the Python package scipy v. 1.8.0 (Virtanen et al. 2020).

## Results and Discussion

As aforementioned, this study has the purpose to exemplify how to apply techniques from the realm of mathematical topology to biological problems, specifically RNA folding, stimulating researches, including students, to think about future research lines inside (or even outside) their area of expertise.

An analysis of the genus of a set of 1147 RNA sequences was performed by means of McGenus software. In particular, the analysis focused on comparing the prediction results from McGenus with topological information extracted from the ‘Genus for biomolecules’ database (based on experimental data), and on investigating the biological significance of RNA structures topologies, both in evolutionary and functional terms. Sequences were phylogenetically diverse and belonging to different types of RNA molecules.

### McGenus Software

Before presenting and discussing the actual results, a brief premise regarding the tool used is necessary. McGenus is a powerful tool and one of the few trying to predict the tertiary structure using, as input, only an RNA sequence in text format. Nevertheless, also due to its age, and despite being written in C to have a higher efficiency of computation, its running time for long sequences is high. As already explained in Sect. 3, it possesses some technical limitations related to the length and ribonucleotide composition of molecules, thus preventing the analysis of some functionally interesting RNA molecules, for example long ribosomal RNA (rRNA) or long non-coding RNA (lncRNA) sequences. Lastly, its functionality is limited, allowing neither for more than a sequence at a time, nor for parallel computations, thus needing the development of Python or R wrappers for more efficient computations and data handling.

**Fig. 6:**
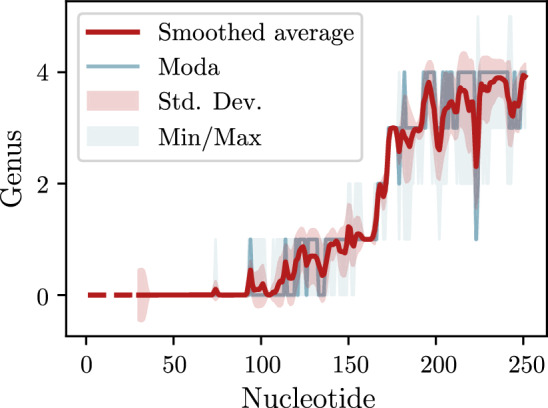
Genus trace, predicted using McGenus, of the RNA sequence with PDB ID 6P5N, chain 1. Average and mode of the 10 suboptimal structures of the statistical ensemble are reported. The same values have been used to shade the area included in the standard deviation (Std. Dev.) area and between the respective minimum and maximum (Min/Max) values. The two main domains of this molecule are clearly visible between ribonucleotides 100-150 and 200-250. Areas of higher variability are an indirect representation of higher structural variability in such area. Average and Std. Dev. have been smoothed for better representation, by applying a 1-D Gaussian filter with sigma equal to 1. Dashed line indicates missing data

Despite these limitations, in addition to using McGenus to calculate the (total) genus of RNA molecules, we were able to show that McGenus can even be used to predict the respective genus traces. We exemplify this concept for the RNA sequence with PDB ID 6P5N (chain 1), which is also present in the ‘Genus for biomolecules’ database (genus trace can be found at https://genus.fuw.edu.pl/view/6p5n/1/). Differently from the genus trace calculated from a 3D structure, the genus trace obtained from McGenus is composed of statistical ensembles, thus having an higher degree of noise, as shown in Fig. 6. Yet, the similarity between the experimental genus trace available at the aforementioned link, and the one computed with McGenus and presented here is striking, clearly showing the two main domains of this molecule. Additionally, McGenus provides extra information, based on the variability of the statistical ensemble, an indirect measure of its structural stability. It is thus clear that McGenus could be exploited also in sight of the most recent developments of the topological concept of genus applied to RNA molecules.

Novel implementations of McGenus algorithm, or alternative ones with the same functions, that circumvent the mentioned limitations, would facilitate the exploration and exploitation of the genus concept in biology. This could have potentially far-reaching consequences in the development of novel ML tools for RNA tertiary structure prediction, or in the design of engineered RNA molecules, or simply in the understanding of the complex structure-function relation within nucleic acids.

### Experimental or Computational Genus?

**Fig. 7:**
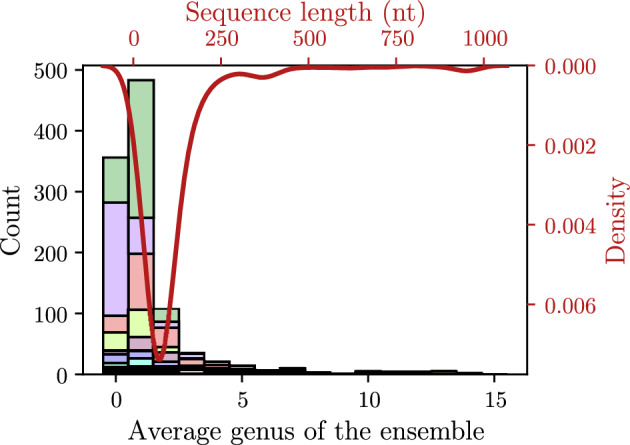
Distribution (*i.e.* kernel density estimation) of sequence lengths (right and top red axes), and histogram (left and bottom black axes) of the predicted genus values obtained with McGenus. Results show how most structures, in the analyzed dataset, are predicted to have a low genus value. This could be due to the majority of sequences being relatively short. Legend has been omitted for sake of space, in the histogram, different RNA types are colored as is Fig. 8(a)

**Fig. 8:**
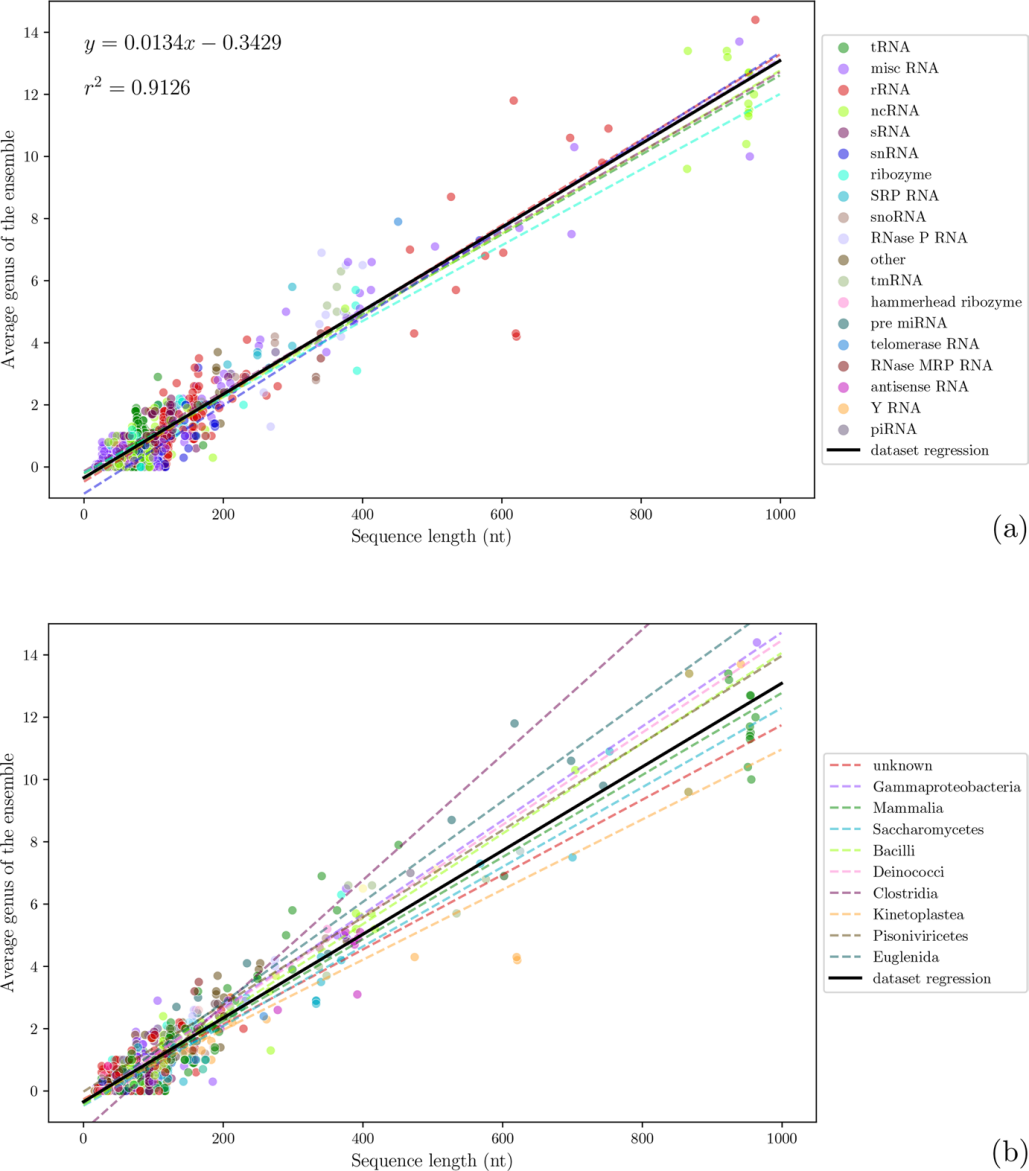
Scatter plots and regression lines of how the predicted average genus compares with the sequences length. The two plots show the exact same data and only highlight differences in distinct types of RNA (**a**) and taxonomic classes (**b**). In (**a**) the ‘dataset regression’ line equation and $r^2$ values are shown; the line is also re-drawn in (**b**) for better comparison. Regression lines for distinct types of RNA and taxonomic classes were only drawn for the most populated groups; for the sake of space, in (**b**), only the most populated groups are reported in the legend

Our dataset mostly consists of relatively short RNA sequences (see Fig. 7). In parallel, the genus predicted for these sequences was on average quite low, with only longer sequences having a higher genus (see Fig. 7 and Fig. 8 (a)). Similarly to Zając et al. (2018), a consistent relationship between sequence length and genus was also found, albeit, even considering only the canonical cWW, the slope of the regression line found computationally in the present paper is more than twice that of the one found experimentally ($g = 0.005d$, see Zając et al. (2018)). Regressing the average genus of the 10 suboptimal structures with the respective sequence length, for the whole dataset of 1147 sequences, yielded that $g = 0.0134d - 0.3429$ (r^2^ = 0.9126, std. err. = 0.00013), where *d* denotes the length of the sequence in ribonucleotides (see the black line in Fig. 8 (a) and its equation). Interestingly, the regression performed using the total genus as calculated in Zając et al. (2018), for the subset of 739 sequences picked for the analysis with McGenus, results in $g = 0.0509d$ (r^2^ = 0.683, std. err. = 0.00128), slightly less than reported in the original publication, while the slope for McGenus predicted values remains basically unchanged ($g = 0.0137$, r^2^ = 0.91, std. err. = 0.00015). Because the total genus calculated using only the canonical cWW is not available (Zając et al. 2018) for the common subset, we could assume that the regression value would be slightly lower, due to the lack of the longer sequences in the dataset, increasing the difference. This would mean that somehow McGenus predictions overestimate the genus when only considering the canonical cWW.

Interestingly, the two above mentioned regression values, *i.e.* the one reported in this study and the one calculated using the total genus from the ‘Genus for biomolecules’ database, are in the same range of those estimated in Li and Reidys (2013). Specifically, Li and Reidys determined, using the central limit theorem, the average genus of random 1-structures (*i.e.* obtained via nesting and concatenating structures of genus at most 1), which results to be 0.091 for $\gamma$-structures of $\tau = 1$ and tend to values closer to 0.013 - 0.015 for $\tau = 6$, and $\gamma = 1$ and $\gamma = 2$ respectively. Here $\tau$ denotes the cardinality of a maximal sequence of “parallel” arcs (*i*, *j*) without intersection between two given labeled nodes *i* and *j*, namely $\#\{(i,j),(i+1,j-1),\dots ,(i+\tau -1,j-\tau +1)\}$.

Looking at the global picture, it is clear that non-canonical pairings play an important role in RNA tertiary structure, and not taking them into account is a major limitation. Yet, even with such a simple analysis, it is noticeable that some sequences present a relatively high or low genus with respect to their length. It would be interesting to understand if there is a biological significance for these deviations from the norm, as it has been demonstrated to be the case for other biological relations such as chromatin contacts frequency (Fudenberg and Mirny 2012). In fact, the frequency of chromatin nucleotides interactions in Euokariotic nuclei follows a distance-dependent decay rule based on their linear distance on the genome (*i.e.* the further away the 2 nucleotides, the lower is the frequency of interaction), but deviations from this relation can be found, and have high biological significance, such as interactions between genetic elements like a promoter and its distal enhancer. Unfortunately, due to the low sample size, it is not currently possible to speculate if a similar concept can be applied to the RNA structures predicted to have a significantly higher or lower genus compared to what would be expected from RNA molecules of their length.

### Exploring Genus (dis)homogeneity

To better understand the possible biological implications of the genus, the differences in distinct types of RNA molecules and taxonomic classes were explored (see Fig. 8a and 8b).

Interestingly, it does not seem that different classes of RNA molecules behave in different ways (see Fig. 8a). Yet, it has to be remarked that while the final dataset included 207 rRNAs sequences, due to the high average length of rRNAs molecules, many publicly available sequences were discarded, as described in Section 3.2. Thus, it would be interesting to see if this behaviour would persist in case of their inclusion in the analysis. Surprisingly, it does not seem that enzymatically active RNA molecules, like the ribozymes, possess a higher or lower genus compared to other RNA molecules like tRNAs, which have a more ‘structural’ role.

On the other hand, looking at the most represented taxonomic classes, there seems to be a more pronounced difference (see Fig. 8b), with some classes having a more steep genus/length relationship. Due to the intrinsic biases present in the dataset used, and since not all taxonomic classes are equally and homogeneously represented, it is hard to draw definitive conclusions.

Yet, despite no clear pattern is visible, as RNA molecules from the three domains of life display an indistinguishable behaviour, it would be interesting to understand whether other factors, *i.e.* environmental conditions, can play a role. Considering there is an intimate relationship between genus and energy, it could be unsurprising to find, for instance, extremophyles to possess an averagely higher genus compared to organisms living at lower temperatures. A given RNA molecule, stable at 37 °C, would for instance be unstable at 80 °C; thus, in a high environmental temperature context, evolution could have favored RNA molecules with a high genus. It has been already demonstrated, in fact, that RNA structure can be phylogenetically conserved even when sequences are not (Caetano-Anolles 2002; Hochsmann et al. 2004; Wuyts 2000). It would be interesting to know whether McGenus predictions would be correct, and thus helpful, by evaluating the results deriving from a set of sequences representing homologs of the same RNA molecule. In this regard, it is important to note that in Zając et al. (2018), authors were able to assign different total genus values to different 3D structures of the same sequence. This is not surprising, since different experimental methodologies can favor different structures and bonds. Unfortunately, McGenus is not able to take into account different conditions, such as, for instance, the presence of ions. These parameters would pose an invaluable addition if integrated into the algorithms presently underlying McGenus.

## Conclusions and Future Perspectives

In this study a useful algorithm for predicting RNA structures based on pseudoknots computation was reviewed again. Firstly, its theoretical background, originally coming from quantum field theory, was explained. Secondly, its practical applications were exemplified by comparing prediction results with experimentally derived data, and by investigating possible biological implications of the topological concept of genus of RNA molecules. Such information can prove of interest and help to students and researcher approaching the topic for the first time. The authors believe that more research efforts should be spent in understanding the technical and biological implications of the concept of genus.

The reviewed and employed McGenus is still an interesting algorithm with a significant potential. It would deserve a suitable update in computational and functional terms, now possible thanks to the last decade of advancements in computer science. Moreover, the newest generalizations of the concept of genus, such as genus trace and genus fingerprint matrix, quoted in Sect. 2.2, could also be integrated as mentioned in Sect. 4.1. This would allow for the prediction of such functions and matrices, while treating the RNA molecule as a statistical ensemble, potentially providing complimentary information than the genus trace of a crystalline and non-dynamic structure.

The results outlined in Sect. 4.3 deserve further analyses, as the preliminary tests show an intriguing behaviour within different taxonomic classes. Previous works proved a relationship between RNA structure and biological function (Caetano-Anolles 2002; Hochsmann et al. 2004; Luwanski et al. 2022; Quadrini et al. 2019; Wuyts 2000), thus similar questions apply to the concept of genus. This should be seen as the first step toward a new research line, which could lead to a more complete understanding of the relationship between the concept of genus and its biological significance, thus improving our comprehension of RNA 2D and 3D structure and its related functions.

In the future, in order to exploit engineered RNA molecules, it will be crucial to be able to predict complex 3D structures starting only from the respective sequences. As briefly outlined in Sect. 1, current methods for either tertiary structure prediction or genus calculation need experimental structural data either to train a ML algorithm (Dykstra et al. 2022; Townshend et al. 2021; Zhang et al. 2022) or for the computation itself (Luwanski et al. 2022; Zając et al. 2018). Having a physics-based, *i.e. ab-initio*, prediction algorithm would be of great assistance in both cases, reducing the necessity of large experimental campaigns, currently needed to collect the necessary data, and thus fostering the development of RNA molecules applications.

Lastly, considering that the algorithm, and cognate theory, underlying McGenus are based on thermo-dynamical properties, it should be possible to expand it to take into account also non-canonical ribonucleotides and, perhaps, specific environmental conditions. Similarly to the introduction of the genus trace concept (Rubach et al. 2019), further exploiting the graph theory could yield additional properties of RNA molecules associated with their respective predictions, although this has yet been demonstrated. As a polymer of ribonucleotides, also RNA molecules are able to store and carry information, and it can be hypothesized that a fraction of such an information can be expressed by the genus and its generalizations. It is thus of interest to understand the presence of a link between the genus and information theory.

Mathematical tools are proving essential to the study of life sciences. For such reason, and to avoid a slowdown of achievements and results, it is crucial for biological researchers and engineers to investigate and evaluate theories and techniques arising from different domains of science (Park et al. 2023). In this view, an update of the algorithm, and the software, based on well-studied mathematical formalism, is compelling. Similarly, there is a necessity for continuous research and development of computational methods borrowed from different scientific realms, for instance from particle physics theories, as exemplified in the present paper.

## Data Availability

The full dataset and results are available as tab separated file at: https://github.com/grassoste/genus-comparisons. McGenus binary files were provided to us by its author Michael Bon. An online version of the software is available at: https://tt2ne.ipht.fr/mcgenus.php

## Footnotes

1 The foundations of this method were developed by physicists between the end of the 70 s and the early 80 s (Bessis et al. 1980; Brézin et al. 1978; ’t Hooft 1974) (QFT). An overview about the applications of Feynman-type diagrams in different scientific domains can be found in Cangiotti (2024).

2 Other two interesting applications of topological techniques for describing a folded molecular chain, namely circuit topology and knot theory, are reviewed in Scalvini et al. (2020).

3 The contemporary formulation of this theory, which explores the graph embedding in manifolds with the aim of finding combinatorial invariants, is due to Alexander Grothendieck. For further details see Grothendieck (1997).

4 Eqs. (1-8) are taken from Vernizzi et al. (2005).

5 For instance, $f(\textbf{r})$ can be taken as a Dirac delta $\delta (|\textbf{r}| - l)$ to model nucleotides connected along the RNA heteropolymer by rigid rods of fixed length *l*.

6 Here we shall use standard mathematical notations, in which $\textrm{d}^{N\times N}\phi$ stands for the volume element of an $N^2$-dimensional integral.

7 A straightforward explanation of this theorem in this context, can be found in Zvonkin (1997).

8 We remark that the matrix dimension *N* serves as an auxiliary parameter that does not correspond to any physical property of the RNA molecule. Rather, it acts as a topological regulator: the $1/N^2$ expansion systematically classifies RNA structures according to their genus (topological complexity of pseudoknots), analogous to the ’t Hooft 1/*N* expansion in large-*N* gauge theories where higher-order terms in $1/N^2$ correspond to increasingly complex topological configurations of the underlying field theory.

9 Sometimes, for computing correctly the values of *V*, *E*, and *F*, it is useful to employ the practical *double line notation* already mentioned.
